# Involvement of GPR17 in Neuronal Fibre Outgrowth

**DOI:** 10.3390/ijms222111683

**Published:** 2021-10-28

**Authors:** Max Braune, Nico Scherf, Claudia Heine, Katja Sygnecka, Thanigaimalai Pillaiyar, Chiara Parravicini, Bernd Heimrich, Maria P. Abbracchio, Christa E. Müller, Heike Franke

**Affiliations:** 1 Rudolf Boehm Institute of Pharmacology and Toxicology, Medical Faculty, University of Leipzig, Härtelstr. 16-18, 04107 Leipzig, Germany; Max.Braune@medizin.uni-leipzig.de (M.B.); claudia.b.heine@t-online.de (C.H.); k.sygnecka@gmail.com (K.S.); 2 Methods and Development Group Neural Data Analysis and Statistical Computing, Max Planck Institute for Human Cognitive and Brain Sciences, Stephanstraße 1A, 04103 Leipzig, Germany; nscherf@cbs.mbp; 3 Department of Pharmaceutical & Medicinal Chemistry, Pharmaceutical Institute, University of Bonn, An der Immenburg 4, 53121 Bonn, Germany; thanigai.medchem@gmail.com (T.P.); christa.mueller@uni-bonn.de (C.E.M.); 4 Department of Pharmaceutical Sciences, University of Milan, Via Balzaretti 9, 20133 Milan, Italy; chiara.parravicini@gmail.com (C.P.); mariapia.abbracchio@unimi.it (M.P.A.); 5 Department of Neuroanatomy, Institute of Anatomy and Cell Biology, Center for Basics in NeuroModulation, Faculty of Medicine, University of Freiburg, Albertstr. 23, 79104 Freiburg, Germany; bernd.heimrich@zfn.uni-freiburg.de

**Keywords:** G protein-coupled receptor 17 (GPR17), neurite outgrowth, montelukast, NG2, *ex vivo* organotypic brain slice co-culture, neurodegeneration and neuroregeneration

## Abstract

Characterization of new pharmacological targets is a promising approach in research of neurorepair mechanisms. The G protein-coupled receptor 17 (GPR17) has recently been proposed as an interesting pharmacological target, e.g., in neuroregenerative processes. Using the well-established *ex vivo* model of organotypic slice co-cultures of the mesocortical dopaminergic system (prefrontal cortex (PFC) and substantia nigra/ventral tegmental area (SN/VTA) complex), the influence of GPR17 ligands on neurite outgrowth from SN/VTA to the PFC was investigated. The growth-promoting effects of Montelukast (MTK; GPR17- and cysteinyl-leukotriene receptor antagonist), the glial cell line-derived neurotrophic factor (GDNF) and of two potent, selective GPR17 agonists (PSB-16484 and PSB-16282) were characterized. Treatment with MTK resulted in a significant increase in mean neurite density, comparable with the effects of GDNF. The combination of MTK and GPR17 agonist PSB-16484 significantly inhibited neuronal growth. qPCR studies revealed an MTK-induced elevated mRNA-expression of genes relevant for neuronal growth. Immunofluorescence labelling showed a marked expression of GPR17 on NG2-positive glia. Western blot and RT-qPCR analysis of untreated cultures suggest a time-dependent, injury-induced stimulation of GPR17. In conclusion, MTK was identified as a stimulator of neurite fibre outgrowth, mediating its effects through GPR17, highlighting GPR17 as an interesting therapeutic target in neuronal regeneration.

## 1. Introduction

Globally, the number of patients dying from and affected by neurological disorders has increased substantially between 1990 and 2015 [1]. In particular, incidence and prevalence of traumatic brain injury (TBI) increased from 1990 to 2016 [2], as well as the prevalence of Parkinson’s disease [3] and dementia [4]. These disorders are the biggest health challenges of the century, posing a serious threat to social and healthcare systems as well as to the future of the global economy [5]. Therefore, new strategies of treatments are pivotal to minimize patients’ disabilities promoting better life quality and to reduce costs for society.

The G protein-coupled receptor 17 (GPR17) has recently been proposed as an interesting pharmacological target in neuroinflammatory and neurodegenerative diseases [6,7,8,9,10,11,12]. The involvement of GPR17 in other pathophysiological conditions of the brain has been demonstrated, leading to the suggestion that GPR17 acts as a ‘sensor of brain damage’ [13]. Under normal physiological conditions, GPR17 is almost exclusively expressed in NG2-glia, also known as oligodendrocyte precursor cells (OPC), which give rise to myelin producing oligodendrocytes both during development and throughout adult life [14]. Own previous data indicate that the GPR17 expression was elevated in human brain specimens from neurosurgical and autoptic cases after TBI [15]. Viganò and co-workers showed, that after cerebral damage induced by acute injury or ischemia in mice, GPR17-positive NG2-glia rapidly reacted to the damage, suggesting these cells are a ‘reserve pool’ of adult progenitors maintained for repair purposes [16]. Subsequent fate mapping studies in the same model of stroke [8] as well as in two different models of demyelination [17] showed that, as a result of injury, GPR17-expressing cells proliferate and markedly accumulate in regions surrounding the lesions, but that only a low percentage of these cells eventually gives rise to mature myelinating oligodendrocytes, due to an unfavourable local inflammatory milieu. These data suggest that GPR17 could be pharmacologically exploited for the benefit of patients with neurological diseases, provided that inflammation is counteracted with appropriate agents.

The pharmacology of GPR17 can be characterized as atypical [18], as GPR17 responds to a diverse set of ligands [9,19,20]. The receptor is phylogenetically related to both purinergic P2Y receptors (P2YRs) and cysteinyl-leukotriene (CysLT) receptors [9]. In 2006, it was discovered that GPR17 is activated by the uracil nucleotides UDP, UDP-glucose and UDP-galactose as well as by the cysteinyl-leukotrienes LTC4 and LTD4 [21]. The known P2Y_12_R antagonists Cangrelor and Ticagrelor and the cysteinyl-leukotriene receptor (CysLT-R) antagonists Montelukast (MTK) and Pranlukast have also been described as antagonists of GPR17 [7,21]. In 2010, Benned-Jensen and Rosenkilde independently confirmed activation of GPR17 by uracil nucleotides [19], while other groups neither found activation of GPR17 by uracil nucleotides or cysteinyl-leukotrienes, nor inhibition of GPR17 by Cangrelor or Ticagrelor [20,22,23]. These varying effects are likely due to the level of expression of GPR17 in the different transfected cell lines utilized, and in the difficulty of preserving the native pharmacological features of the receptor in artificial recombinant systems.

It has been demonstrated that in animal models of stroke treatment with Cangrelor, and also with MTK and blockade of GPR17 with antisense technology, led to decreased infarct volumes [13,21,24,25]. In rats, treatment with MTK reduced neuroinflammation, elevated hippocampal neurogenesis and improved learning and memory [26]. These effects were mediated by GPR17 as demonstrated by using gene knock down and knock out strategies [26].

Otherwise, in PC12 cells, that natively express GPR17, treatment with UDP-glucose and LTD4 promoted survival and neurite outgrowth [27]. In a neonatal rat model of ischemic periventricular leukomalacia treatment with UDP-glucose improved the thickness of myelin sheaths, motor dysfunction and cognitive functions [28].

Due to its special pharmacology and distinct results, the role of GPR17 in neuroreparative and neuroregenerative mechanisms has not yet been completely elucidated. In particular, it is not clear if and how signalling of GPR17 is involved in repair mechanisms in projection systems after trauma and whether these can be influenced by treatment with ligands of GPR17.

For this reason, the well-established rat *ex vivo* model of organotypic slice co-cultures of the mesocortical dopaminergic system was chosen culturing slices of the substantia nigra/ventral tegmental area (SN/VTA) and the prefrontal cortex (PFC) closely together allowing neuronal fibre outgrowth, to grow from one region to the other [29,30]. Organotypic slice co-cultures largely preserve the tissue architecture of the brain regions that they originate from [31], thereby modelling the *in vivo* situation closely [32]. While the preparation of the co-cultures causes a disruption of the already established connections, this model also strongly correlates with the development of fibre projections of physiological neuronal circuits, thus making the organotypic co-cultures a model of both development and of axonal regrowth after mechanical injury [30]. Concretely, in the present study it was examined (i) whether pharmacological stimulation or inhibition of GPR17 can promote neurite outgrowth; (ii) the dynamics of a small selection of genes involved in growth and differentiation of neurons, myelination and inflammation depending on the treatment with GPR17 antagonists and agonists; (iii) the temporal expression of GPR17 in the co-cultures; and (iv) which cells might be effectors of any observed effects.

## 2. Results

### 2.1. Neurite Fibre Outgrowth Modulation

**Figure 1:**
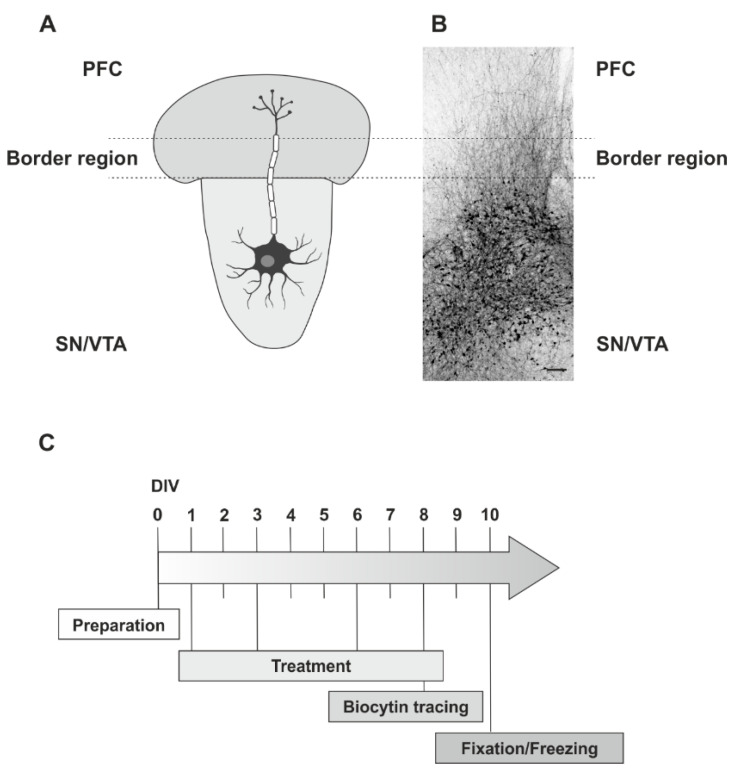
Experimental design. (**A**) Schematic Illustration of the used organotypic *ex vivo* slice co-cultures of the dopaminergic system (SN/VTA and PFC) (Attribution: Free neuron vector from vecteezy: Available online: https://www.vecteezy.com/free-vector/nerve-cell (accessed on 23 July 2021). Nerve Cell Vectors by Vecteezy. (**B**) Overview of the fibre outgrowth visualized by biocytin tracing in an *ex vivo* co-culture system (rat, fixed at DIV 10). The dotted lines characterize the border region, used for quantification of fibre density. (**C**) Timeline with the treatment procedures. Scale bar: **B** = 200 µm.

To elucidate if modulation of the GPR17 has any effects on neurite outgrowth in organotypic dopaminergic co-cultures the well-established neurite fibre quantification method in our lab was used [33] (for schematic illustration see Figure 1).

In initial experiments, the effect of Cangrelor (an anti-platelet agent blocking the P2Y_12_Rs, and reported to act additionally as a non-selective GPR17 antagonist) was investigated, and a significant stimulatory effect on neurite fibre outgrowth in comparison to ACSF (*t*-test with Welch correction t (4.34607) = −3.28534; *p* = 0.02685; see Figure S1) was observed.

**Figure 2:**
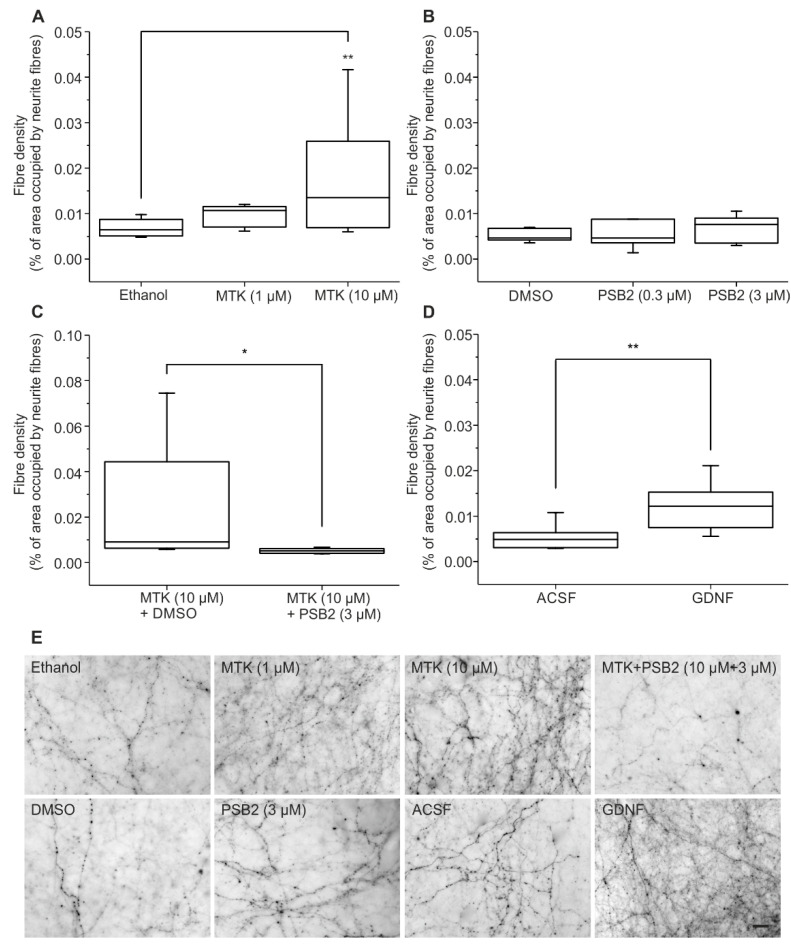
Neurite outgrowth quantification. (**A**–**D**) Neurite fibre density was quantified after treatment with (**A**) MTK, (**B**) PSB-16484 (PSB2), (**C**) MTK and PSB-16484 (PSB2) and (**D**) GDNF using biocytin-tracing. Data are shown as box plots. The number *n* of animals being used was for (**A**) *n* ≥ 9, (**B**) *n* = 4, (**C**) *n* ≥ 5, (**D**) *n* = 8. For (**A**,**B**) ANOVA on Ranks was followed by Tukey’s test, for (**C**) Mann–Whitney test was applied and for (**D**) *t*-test was applied; * *p* < 0.05, ** *p* < 0.01. (**E**) Pictures of biocytin-labelled fibres in the border region after application of Ethanol, MTK (1 µM), MTK (10 µM), MTK (10 µM, pre-treatment) + PSB-16484 (PSB2, 3 µM), DMSO, PSB-16484 (PSB2, 3 µM), ACSF and GDNF. Scale bar: 20 µm for all.

In the next phase of experiments, different concentrations of MTK, the CysLT1R antagonist and proposed GPR17 antagonist, were employed. In the dopaminergic slice co-cultures, in a similar way to Cangrelor, the application of 10 µM of MTK showed a significant stimulation of neurite fibre outgrowth (Figure 2A,C) compared to vehicle (A: 1% ethanol; C: 0.01% DMSO, see Section 4.4.) treated controls (one-way ANOVA F (2,28) = 6.143; *p* = 0.00614; followed by Tukey’s post hoc test *p* = 0.0062). Neurite fibre outgrowth after application of 1 µM MTK was tendentially increased, but not significantly (Tukey’s post hoc test *p* = 0.57299; Figure 2A).

Recently, the small molecule 3-(2-carboxy-4,6-dichloro-indol-3-yl)-propionic acid (MDL29,951) was identified to act as a GPR17 agonist [7,20], and optimized analogues of MDL29,951 (which is non-selective since it also interacts with NMDA receptors), were synthesized [34,35] and characterized in functional assays. In a radioligand binding assay, affinity of this class of compounds for GPR17 was confirmed and blockade by MTK was shown [20,34]. Hence, two of the new, selective GPR17 agonists were used to activate the receptor, namely PSB-16282 and PSB-16484, while MTK was employed as a GPR17 antagonist.

The application of the new GPR17 agonists alone, PSB-16484 (PSB2) (one-way ANOVA F (2,19) = 0.29054; *p* = 0.75113) (Figure 2B) and PSB-16282 (PSB1) (see Figure S2 (one-way ANOVA F (2,12) = 1.04803; *p* = 0.38063)), did not cause a significant increase or decrease in neurite fibre outgrowth. Thus, for additional studies, to reduce use of animals only PSB-16484 (PSB2) was utilized.

To evaluate if the observed stimulatory effect of MTK could be antagonized by a new, selective synthetic GPR17 agonist, 10 µM MTK was applied in combination with 3 µM PSB-16484 (PSB2) and compared to a group treated only with 10 µM MTK. Neurite fibre density of cultures treated with MTK and PSB-16484 (PSB2) was significantly lower compared to co-cultures treated only with MTK (Mann-Whitney-Test U = 28; *p* = 0.01732; Figure 2C) indicating that the stimulation of neurite fibre outgrowth after application of MTK is, at least in part, mediated by GPR17.

As a positive control for stimulation of neurite fibre outgrowth, the well-established neurotrophic factor GDNF, which has been shown to provide neuroprotective and neurotrophic effects especially with regard to the mesencephalon, was used [36]. The application of GDNF (50 ng/mL) induced a significant stimulation of neurite fibre outgrowth (*t*-test t (14) = −3.34886; *p* = 0.00477) compared to controls, proving its potency as a neurotrophic factor and demonstrating that neurite fibre outgrowth could be stimulated (Figure 2D).

In comparison, treatment with 10 µM MTK showed a 2.6-fold higher mean neurite density compared to controls, while treatment with GDNF led to a 2.3-fold higher mean neurite density compared to controls suggesting that 10 µM MTK is a notable stimulator of neurite fibre outgrowth in the organotypic dopaminergic co-culture system. Examples of neurite fibre outgrowth are given in Figure 2E.

### 2.2. Gene Expression Analysis of Genes Relevant for Neuronal Growth

**Figure 3:**
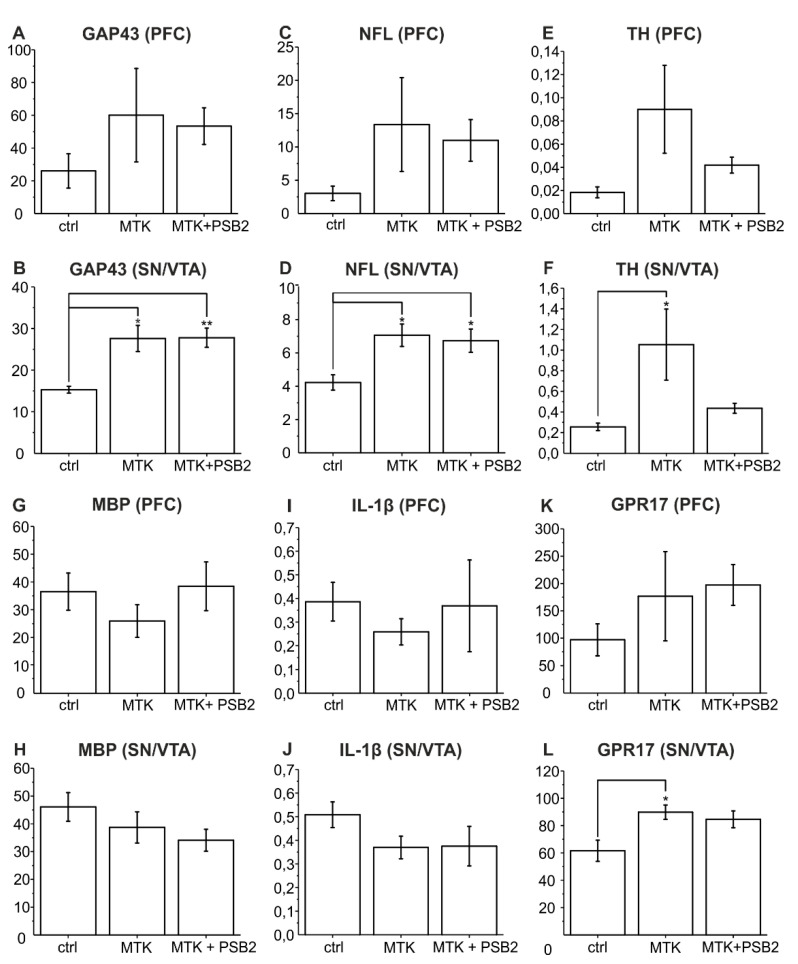
Results of RT-qPCR experiments in the PFC and SN/VTA. Expression of mRNA of GAP43 (**A**,**B**), NFL (**C**,**D**), TH (**E**,**F**), MBP (**G**,**H**), IL-1β (**I**,**J**), GPR17 (**K**,**L**) is shown on *y*-axis as ∆ CP. X-Axis represents the different treatments (1% ethanol and 0.01% DMSO as controls (ctrl); 10 µM MTK and 0.01% DMSO; 10 µM MTK and 3 µM PSB-16484 (PSB2)). Statistical analysis was performed in comparison to vehicle control with ANOVA on ranks followed by Tukey‘s test. Data are shown as bar charts. The number of animals being used was *n* = 4, * *p* < 0.05, ** *p* < 0.01.

To get further insight into the effects caused by application of MTK and PSB-16484 (PSB2), a gene expression analysis was performed using reverse transcription quantitative real-time polymerase chain reaction (RT-qPCR) after day 10 in culture. In addition to GPR17, the expression of five target genes representative of functions in the central nervous system (CNS) or specific cell populations (Figure 3: PFC (A,C,E,G,I,K); SN/VTA (B,D,F,H,J,L)) (Table 1) were investigated.

GAP43 and NFL were chosen because of their role in axonal regeneration [37] and as regeneration associated genes [38]. In brief, GAP43 is known as being connected to neuronal growth, promoting spontaneous formation of new synapses and enhancing sprouting after injury. Additionally, it is linked to neurite outgrowth, nerve-terminal sprouting and long-term potentiation [39]. Its expression in the used *ex vivo* slice co-cultures has been shown immunohistochemically [30]. In the present study, the expression of mRNA of GAP43 (Figure 3A,B) was significantly elevated in the SN/VTA after treatment with MTK compared to controls (ctrl) (one-way ANOVA F (2,9) = 9.73918; *p* = 0.00561; followed by Tukey’s post hoc test *p* = 0.01067; Figure 3B) supporting the findings in neurite density quantification. Application of PSB-16484 (PSB2) could not block this effect (Tukey’s post hoc test *p* = 0.99838). There was no significant difference between groups in the PFC (one-way ANOVA F (2,9) = 0.92958; *p* = 0.42954; Figure 3A).

NFL is particularly abundant in axons where it is essential for the radial growth of axons during development, the maintenance of axon calibre and the transmission of electrical impulses [40]. Our results show that mRNA-expression of NFL (Figure 3C,D) was significantly stimulated by treatment with MTK in the SN/VTA (one-way ANOVA F (2,9) = 6.23136; *p* = 0.02002; followed by Tukey’s post hoc test *p* = 0.02547; Figure 3D) supporting our findings of higher neurite density in the border region between PFC and SN/VTA after treatment with MTK. The MTK effect was not reduced by PSB-16484 (PSB2) (Tukey’s post hoc test *p* = 0.92805). There was no significant difference between groups in the PFC (one-way ANOVA F (2,9) = 1.45291; *p* = 0.28391; Figure 3C).

It should be evaluated, if dopaminergic neurons benefit from application of MTK in the co-culture. Therefore, measurement of the mRNA-expression of TH, the marker enzyme of dopaminergic neurons, was performed [41]. TH mRNA-expression (Figure 3E,F) was significantly elevated in the SN/VTA after treatment with MTK (one-way ANOVA F (2,9) = 4.29564; *p* = 0.04901; followed by Tukey’s post hoc test *p* = 0.0498) and showed non-significantly reduced expression after treatment with PSB-16484 (PSB2) (Tukey’s post hoc test *p* = 0.13171; Figure 3F). Again, in the PFC there was no significant difference between groups (one-way ANOVA F (2,9) = 2.65474; *p* = 0.1241; Figure 3E).

Additionally, gene expression of myelin basic protein (MBP) was analysed. MBP is an essential part of the myelin sheath insulating axons electrically and thereby allowing saltatory conduction and high conduction velocity [42]. Since GPR17 has been shown to play a role in oligodendrocyte maturation [43,44], it was hypothesized that application of MTK and PSB-16484 (PSB2) could affect expression of the key oligodendrocyte marker MBP. However, expression of mRNA of MBP (Figure 3G,H) showed no significant change after treatment with MTK and PSB-16484 (PSB2) in the PFC (one-way ANOVA F (2,9) = 0.867; *p* = 0.45255; Figure 3G) and the SN/VTA (one-way ANOVA F (2,9) = 1.48997; *p* = 0.27609; Figure 3H).

Furthermore, the question was addressed if the observed neuroregenerative effects of MTK could be assigned to a decreased neuroinflammatory milieu in the dopaminergic co-cultures. Because of its central role in neuroinflammation, IL-1β was chosen as an appropriate marker [45]. However, mRNA-expression of IL-1β (Figure 3I,J) showed no significant changes after treatment with MTK and PSB-16484 (PSB2) but was non-significantly decreased in the PFC (one-way ANOVA F (2,9) = 0.30249; *p* = 0.7462; Figure 3I) and the SN/VTA after treatment with MTK (one-way ANOVA F (2,9) = 0.45195; *p* = 0.65008; Figure 3J). Treatment with PSB-16484 was able to alleviate this tendency in the PFC, but not in the SN/VTA.

Finally, the expression of mRNA of GPR17 (Figure 3K,L) was monitored, which was found to be significantly elevated after treatment with MTK in the SN/VTA (one-way ANOVA F (2,9) = 5.36593; *p* = 0.02923; followed by Tukey’s post hoc test *p* = 0.03199), but not in the PFC (one-way ANOVA F (2,9) = 0.94476; *p* = 0.42418; Figure 3K,L). Treatment with PSB-16484 (PSB2) led to no significant difference in expression of GPR17 compared to treatment with MTK in the PFC (Tukey’s post hoc test *p* = 0.96131) and the SN/VTA (Tukey’s post hoc test *p* = 0.83958).

In conclusion, the data show that the mRNA-expression of all three neuronal markers (GAP43, NFL, TH) as well as of GPR17 are significantly increased after treatment with MTK in SN/VTA, but not in the PFC, supporting a neuroregenerative effect after treatment with MTK and suggesting regional differences in responsiveness to MTK.

### 2.3. Dynamics of GPR17 Expression in Untreated Organotypic Dopaminergic Co-Cultures

#### 2.3.1. RT-qPCR Analysis of GPR17 Expression

**Figure 4:**
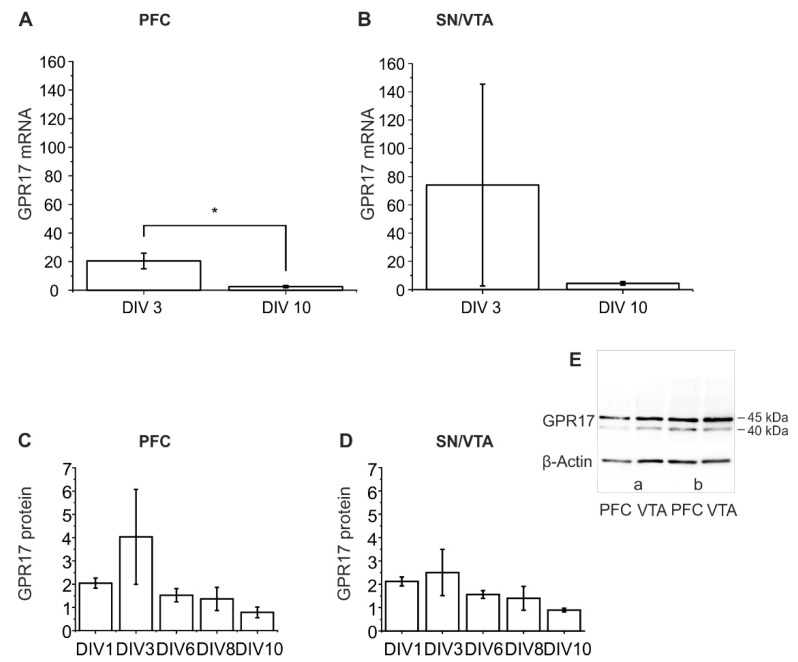
Results of RT-qPCR and Western blot of GPR17 in the *ex vivo* co-cultures. Expression of mRNA of the GPR17 is shown as ∆ CP in the PFC (**A**) and SN/VTA (**B**) after DIV 3 and DIV 10. Statistical analysis was performed in comparison to vehicle control with ANOVA on ranks followed by Tukey‘s test. Data are shown as bar charts. The number of animals was *n* = 3, * *p* < 0.05. Protein expression of GPR17 is shown on DIV 1, 3, 6, 8 and 10 in PFC (**C**) and SN/VTA (**D**), data are shown as bar charts. The number of animals being used was *n* ≥ 3. For statistical analysis one-way ANOVA (PFC) and Kruskal–Wallis (SN/VTA) was used. (**E**) Representative examples of Western blot images of samples obtained (DIV 10; (a) ctrl (Ethanol); (b) MTK 10 µM).

The mRNA expression of GPR17 was characterized at two time points, DIV 3 and DIV 10. In both regions, PFC and SN/VTA, a higher expression was found on DIV 3. The expression of mRNA of GPR17 was significantly reduced after DIV 10 compared to DIV 3 in the PFC (*t*-test t (4) = 3.22794; *p* = 0.03204) and tendentially reduced in SN/VTA (*t*-test with Welch correction t (2.00119) = 0.97405; *p* = 0.43272; Figure 4A,B). This corresponds well to the Western blot results, also pointing to an increase in expression of the GPR17 caused by the preparation (injury-induced increase in GPR17 mRNA) (Figure 4C,D).

#### 2.3.2. Western Blot Analysis

Using the rabbit anti-GPR17 antibody (Sigma-Aldrich, St. Louis, MO, USA), two protein bands were labelled, one at 40 kDa and one at 45 kDa (Figure 4E). These data are in agreement with previously published literature data suggesting that the band at 40 kDa could be a precursor form carrying high mannose oligosaccharide chains [46]. Western blot analysis showed that GPR17 had its peak of expression both in PFC and SN/VTA after DIV 3, followed by a subsequent decrease (Figure 4C,D). There were no significant differences between DIV in the PFC (one-way ANOVA F (1.69403), *p* = 0.22724) and the SN/VTA (Kruskal–Wallis ANOVA chi-square (4) = 6.47059; *p* = 0.16665).

Furthermore, for comparison, native tissue taken from young rats (P2 and P13), from both regions, PFC and SN/VTA, (Figure S3) was investigated. The data showed a slight, but not statistically significant increase in the expression of GPR17 at age P13 compared to P2 in PFC (*t*-test with Welch correction t (3.81358) = −0.62477; *p* = 0.56755) and SN/VTA (*t*-test t (4) = −0.15674; *p* = 0.88304).

In conclusion, the data suggest an injury-induced GPR17 expression in the studied *ex vivo* model. Under *in vivo* conditions no significant changes in GPR17 protein expression between day 2 and day 13 were found (no developmental increase in the investigated regions). An injury-induced (slice preparation-induced) increase at DIV 3 was postulated. These data are supported by previous experiments using tissue of the complete co-culture (PFC and SN/VTA), indicating an increase at DIV 3 too (data not shown).

### 2.4. Immunohistochemical Analysis of GPR17 Expression

To investigate which cells express GPR17 in the organotypic dopaminergic co-cultures and could therefore be targeted with ligands of GPR17, the immunolabelling of GPR17 and characteristic cell makers was performed (Table 2).

**Figure 5:**
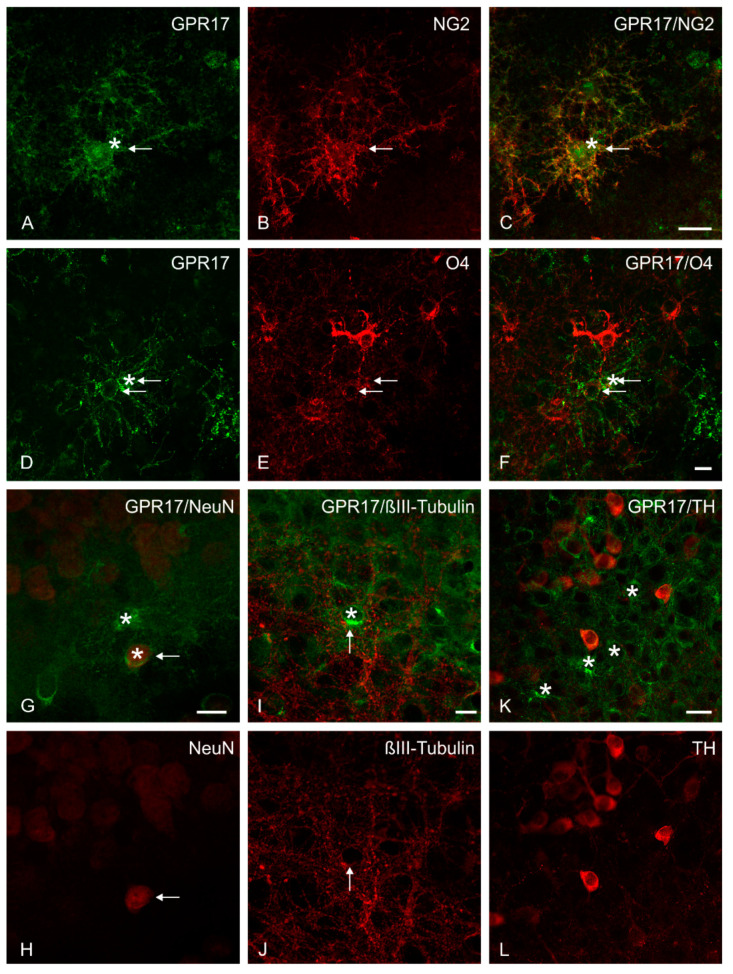
Multiple immunofluorescence study. Representative confocal images of GPR17 expression in organotypic slice co-cultures. At DIV 10 an intense GPR17 immunoreactivity was observed on NG2-positive cells (**A**–**C**) and on O4-positive cells (**D**–**F**). A low expression of GPR17 (stars) on a small number of cells was observed on (**G**,**H**) NeuN-positive cells and (**I**,**J**) βIII-Tubulin-positive neurons (the thin arrows indicate the co-expression). No co-localization, but a number of GPR17-positive cells (stars) in the proximity were found on (**K**,**L**) TH-positive cells. Scale bars: (**A**–**C**) = 20 µm; (**D**–**J**) = 10 µm; (**K**,**L**) = 20 µm.

The results show that GPR17 is mainly expressed by NG2-positive glia (Figure 5A–C), but also by neurons (low labelling on NeuN-positive cells; examples are given in Figure 5G,H).

GPR17 is rarely expressed by more developed cells of the oligodendroglial cell lineage (O4-, CNPase-positive cells; an example for O4 is given in Figure 5D–F) and very low in MBP-positive cells, reflecting the peculiar time dependent expression of this receptor during oligodendrocyte maturation [47]. GPR17-labelled cells were observed rather in the proximity of MBP-positive structures (Figure S4A–C). There is also weak expression on microglia (Iba1-positive cells, Figure S4D–F), but no expression on astrocytes (GFAP-positive cells). Other neuronal markers showed only weak (e.g., βIII-Tubulin-positive cells) or no (TH-positive, NFL-positive cells) co-localization with GPR17 (examples are given in Figure 5I,J,K,L). It is important to note that GPR17-positive cells (stars) could be found more in direct proximity of βIII-Tubulin-, TH- and NFL-positive neurons, but not co-expressed on the TH- or NFL-positive structures. It was hypothesized that GPR17 expression was induced in neurons of our co-cultured slices as a result of the experimental procedure, and reflects a neuronal response to the trauma of the slice preparation.

### 2.5. Toxicological Analysis

**Figure 6:**
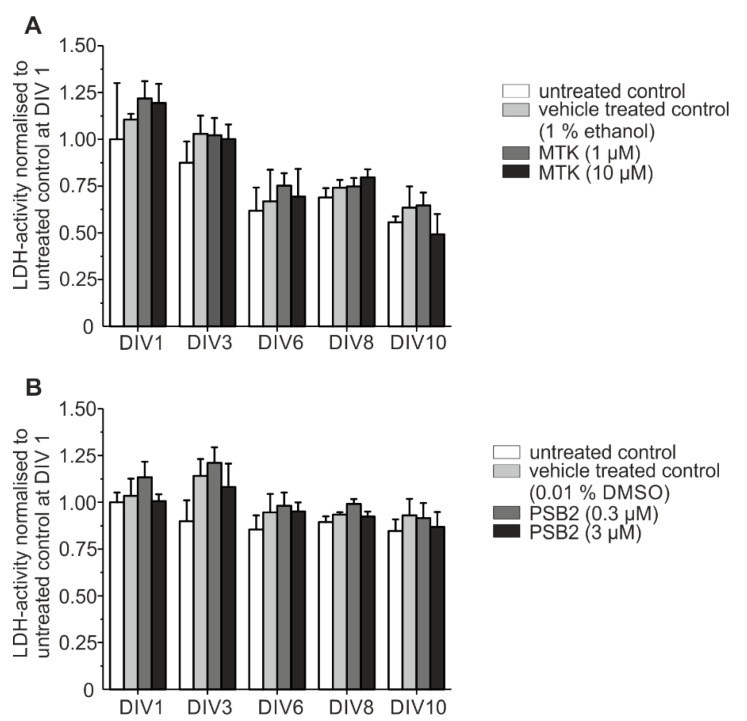
Toxicity testing. (**A**) LDH activity was measured after treatment with MTK (**A**) and PSB-16484 (PSB2) (**B**) compared to vehicle treated control und untreated control. Data have been normalized to the control value at DIV 1 of the respective preparation. Statistical analysis was performed using repeated measurement two-way ANOVA. Each sample represents incubation medium (IM) of one animal. The number of animals was *n* = 5.

To ensure that the applied pharmacological substances had no toxic effects on the organotypic slice co-cultures, the LDH release into incubation medium (IM) was measured. Data showed no significant elevation of LDH activity after treatment with 1 µM and 10 µM MTK compared to untreated control and vehicle-treated control (1% ethanol or 0.01% DMSO; repeated measurement two-way ANOVA with Greenhouse–Geisser correction F (0.29814, 0.60849) = 1.95985; *p* = 0.2293) e.g., 1% ethanol; Figure 6).

After treatment with PSB-16484 (PSB2) the LDH activity was neither elevated compared to untreated control nor to vehicle-treated control (repeated measurement two-way ANOVA with Greenhouse–Geisser correction F (1.6208, 6.48319) = 4.87266; *p* = 0.05565) 0.01% DMSO). Overall LDH activity decreased from DIV 1 to DIV 10 with no significant differences between pharmacological treatment groups and vehicle-treated controls. These data provide proof that neither MTK nor PSB-16484 (PSB2) is toxic to the organotypic dopaminergic slice co-cultures (Figure 6). Both effects, (i) the increase in LDH release (representing the mechanical lesion as a result of the preparation of the co-cultures) and (ii) the decrease in LDH release in the following DIV agrees very well with previous data from our group [48].

### 2.6. Electron Microscopy

The electron microscopic observation revealed the presence of multi-lamellar myelin-like structures in the slice co-cultures of SN/VTA at DIV 10 of incubation (Figure S5A,B) and also in the early postnatal (P12) ventral midbrain (Figure S5C). High resolution images display n-like lamellar organized membranous structures in the SN/VTA part of the co-culture. Additionally, in the developing fibres bridge from SN/VTA to the PFC, irregularly organized myelin-like lamellae were found ensheathing neuronal profiles (Figure S5B).

For comparison, in the ventral midbrain (P12, rat) cross-sectioned axons of varying diameters could be detected. Only very few axons were enveloped by membranous myelin-like sheaths (Figure S5C).

## 3. Discussion

The present results confirm the ability of MTK to promote axonal outgrowth and suggest the involvement of GPR17 in these effects. In more detail, the obtained data in the dopaminergic organotypic slice co-cultures indicate that (i) MTK, a non-selective GPR17 antagonist, can promote neurite outgrowth with effects comparable to those induced by the well-known neurotrophic factor GDNF, (ii) treatment with MTK increases mRNA-expression of genes relevant to neuronal growth, (iii) a clear expression of GPR17 on NG2-glia and in some NeuN-positive neurons, (iv) a time-dependent expression of GPR17 in untreated organotypic dopaminergic co-cultures. These results could be of special interest to patients with TBI and other neurological disorders, suggesting that MTK possibly not only attenuates damage, but also promotes neuroregeneration and repair.

### 3.1. Pharmacological Inhibition of the GPR17 with MTK Can Promote Neurite Outgrowth

The present data show that in the studied dopaminergic organotypic slice co-cultures neuronal outgrowth was stimulated by treatment with the CysLT1-R antagonist and GPR17 antagonist MTK, with effects comparable to those induced by the well-known neurotrophic factor GDNF.

In order to determine if this effect occurred by antagonism of GPR17, two new synthetic GPR17 agonists were applied alone to the co-cultures showing no stimulatory but also no inhibitory effect on neurite outgrowth. Possibly, this reflects the release of endogenous agonists of GPR17 in the slice co-cultures following the preparation procedure, making it difficult to reveal an additional effect caused by the synthetic agonist [49]. Instead, the role of GPR17 was unveiled when the GPR17 agonist PSB-16484 was applied to cultures in the presence of MTK, as shown by abrogation of the MTK induced neurite outgrowth, supporting inhibition of GPR17 as an essential mechanism at the basis of the neuroregenerative effect of MTK under these conditions.

Previous studies in PC12 cells had shown an enhanced neurite outgrowth after treatment with the proposed GPR17 agonist UDP-glucose [27]. While UDP-glucose might have a growth-supporting effect on neurites, its activity as a GPR17 agonist has been questioned [20,22,23]. The different outcomes could also be a result of the chosen models investigating neurite outgrowth, as the organotypic slice co-cultures preserve the architecture and microenvironment of the brain with all its cellular players [30] in contrast to the PC12 cell culture model.

Our initial experiments indicated a growth-promoting effect of the employed P2Y_12_R and proposed GPR17 antagonist Cangrelor, which may support a beneficial effect of GPR17 antagonists on neurite outgrowth. However, the effect of Cangrelor (playing a central role in the complex processes of activation and aggregation in blood platelets) [50] may be indirect, involving modulation (inhibition) of inflammatory processes by blocking P2Y_12_Rs e.g., on microglial cells [50,51,52].

As the specificity and validity of some ligands of GPR17 have been questioned [23,53] the use of new synthetic ligands of GPR17 is a promising way to unveil its effects. A number of recently published studies support the role of MTK as a GPR17 antagonist. MTK was found to inhibit a synthetic GPR17 agonist [^3^H]2-carboxy-4,6-dichloro-1*H*-indole-3-propionic acid ([^3^H]PSB-12150) in a concentration-dependent manner [20]. Furthermore, it was shown that MTK enhanced the growth of neurospheres due to blockade of GPR17 and that knockout of GPR17 led to increased proliferation of neurospheres [26].

### 3.2. MTK Promotes Neurite Outgrowth by Elevating Neurotrophic Gene Expression

In order to gain some insight into the downstream effects following treatment of the slice co-cultures, RT-qPCR was used to investigate genes involved in neuronal growth of dopaminergic neurons, inflammation and myelination.

The presented RT-qPCR results support a growth-promoting effect of MTK in the co-cultures as mRNA-expression of GAP43, NFL and TH is significantly elevated after treatment with MTK in SN/VTA. GAP43 is a regeneration associated gene [38] and overexpression of GAP43 has been shown to lead to spontaneous formation of new synapses and enhanced sprouting after injury [39]. By stimulating expression of GAP43, treatment with MTK could promote sprouting of neurites. The neurofilament NFL is essential for the radial growth of axons [40]. Treatment with MTK could thus lead to enhanced axonal growth. TH is known to be the key enzyme of dopaminergic neurons and has been used as the characteristic marker for developing dopaminergic fibres from the SN/VTA to the PFC [29,41]. Elevated expression of TH after treatment with MTK underlies MTKs potency to promote growth of dopaminergic neurons in the slice co-cultures.

Therefore, a neurogenic effect of MTK is conceivable as this would also be in line with previously published literature. Transcriptome analysis has shown that GPR17 is specifically highly expressed in adult neural progenitor cells [54]. Recent studies showed that blockade of GPR17 with MTK led to elevated neural stem and progenitor cell proliferation [26,55]. Treatment with MTK resulted in improved cognition of old rats correlating best with enhanced neurogenesis [26]. GPR17 knockdown and knockout in neurospheres induced hyperproliferation and abolished the effects of montelukast [26].

Summarizing, MTK promotes neurite outgrowth in the dopaminergic slice co-culture by enhancing gene expression of neurotrophic genes like GAP43 and NFL. Elevation of mRNA of TH in the SN/VTA after treatment with MTK supports the observed stimulatory effect of neurite fibre outgrowth in the fibre density quantification.

Our data also indicate that GPR17 is not the only mediator of MTKs neurotrophic effects, since MTK induced expression of NFL and GAP43 could not be lowered by treatment with the GPR17 agonist PSB-16484. Only the stimulated mRNA expression of TH was tendentially lowered when co-cultures were treated with PSB-16484, suggesting that elevation of TH could be specifically caused by targeting GPR17 and implying an especially beneficial effect for dopaminergic neurons after blocking GPR17.

### 3.3. Is Neurite Outgrowth by MTK Stimulated by Modulation of Neuroinflammatory Pathways?

The recent literature data from *in vitro* and *in vivo* experiments suggests that GPR17 is a sensor of damage [13]. It was shown that MTK reduces neuroinflammation, elevates hippocampal neurogenesis and improves learning and memory in 20-month-old rats [26]. By using gene knockdown and knockout approaches, this effect was demonstrated to be mediated through inhibition of GPR17 reducing microglial activation and elevating neurogenesis [26]. After traumatic brain injury in the human brain an increased expression of GPR17 was found followed by a decrease within a few days [15]. In the present study, in untreated dopaminergic *ex vivo* co-cultures, expression of GPR17 was high after DIV 3 in RT-qPCR and Western blot results possibly responding to the tissue damage inflicted by the preparation process with upregulation of GPR17. The Western blot data on native (uninjured) tissue (P2, P13) do not show this effect suggesting that GPR17 expression is a time-dependent dynamic parameter in our model possibly responding to the trauma caused by the preparation of the co-cultures and comparable with the *in vivo* data after TBI.

Inflammatory processes following the preparation procedure (cutting tissue) are conceivable. The authors hypothesized that by treating co-cultures with MTK, a reduction in these neuroinflammatory processes could lead to increased neurite outgrowth. In order to investigate a possible role for neuroinflammatory modulation the mRNA-expression of IL-1β was examined which is known to play a central role in mediating neuroinflammation in pathologies of the CNS [45]. However, in the present study after DIV 10 and treatment with MTK there was only a tendency of reduced expression of mRNA of IL-1β in the dopaminergic slice co-cultures. In the PFC this tendency seemed to be revoked by treatment with PSB-16484 suggesting involvement of GPR17, but differences between the groups were not statistically significant. It cannot be excluded that significant effects on expression of IL-1β measured earlier than on DIV10 might have been missed.

Interestingly, a co-localization of GPR17 with the microglial marker Iba1 was observed. Microglia are known to contribute to neuronal death and neurodegenerative pathologies [56], and microglial inhibition has been shown to be neuroprotective [57]. In a previous study, it has furthermore been demonstrated, that GPR17 mediates ischemia-like neuronal injury via microglial activation [58]. Recently, it was shown that chronically activated microglia and signalling of GPR17 inhibit maturation of OPC and myelination in an optic nerve injury model [59].

Thus, microglial expression of GPR17 and recent literature support an anti-inflammatory effect of treatment with GPR17 antagonists. The known activity of MTK as a CysLTR1 antagonist suggests a role for anti-inflammatory effects. However, in the organotypic slice co-culture model, significant effects on mRNA-expression of IL-1β after treatment with MTK reflecting anti-inflammatory activity were not confirmed but might have been missed. Hence, a relevant anti-inflammatory effect of treatment with MTK cannot be excluded. Additionally, the effect of Cangrelor may partly be explained by an inhibitory role in microglial activation via P2Y_12_Rs as described above.

### 3.4. Is Neurite Outgrowth Stimulated by Targeting Oligodendrocytes in the Co-Culture?

Oligodendrocytes produce myelin which allows for saltatory impulse propagation, but they also exert important trophic functions for neurons [60,61,62]. GPR17 plays an important role in oligodendrocyte differentiation [43,47] and was shown to be a cell-intrinsic timer of myelination [6]. Expression of GPR17 is necessary to start oligodendrocyte differentiation, but must then be downregulated to allow terminal cells’ maturation and myelination [18,63,64]. Recently, it was demonstrated that treatment with MTK in a mouse model of stroke increased expression of MBP, numbers of oligodendrocytes and fibre connectivity [65].

On this basis, the expression of GPR17 in the organotypic slice co-culture model using immunohistochemistry was examined. As expected, expression of GPR17 was most abundant on NG2-glia. NG2-glia are OPCs responsible for the generation of mature oligodendrocytes during development and adulthood [14,47].

There is also evidence for NG2-glia maintaining neuronal functions and survival of neurons through regulation of neuroinflammatory pathways [66] and it was shown that NG2-glia are permissive to neurite outgrowth and stabilize sensory axons [67]. Furthermore, participation of the GPR17 to post-acute reactivity of NG2-glia in different injury paradigms was demonstrated [68]. It has been suggested that treatment of NG2-glia with GPR17 ligands could possibly influence their differentiation potential or activate reparative functions [16].

In other models of neurodegeneration, an abnormal increase in GPR17 has invariably been associated with myelin defects and its pharmacological manipulation succeeded in restoring endogenous remyelination. Furthermore, OPCs (isolated from spinal cord of SOD1^G93A^ mice) display defective differentiation compared to control cells, which is rescued by treatment with the GPR17 antagonist MTK [12]. It is concluded that, as a result of either acute injury (e.g., stroke, trauma or demyelination) or genetic defects (as is the case of SOD1^G93A^ mice), GPR17 is initially induced to promote OPC maturation, but its persistence in cells and inability to undergo down regulation unfavourably affects cell’s terminal maturation and myelination.

Under these conditions, pharmacological antagonism of GPR17 with MTK alleviates this maturation block, and enables OPCs to resume differentiation, which would in turn possibly promote neurite outgrowth. However, mRNA expression of MBP was not enhanced after treatment with MTK on DIV 10, suggesting that the observed effects of MTK on neurite outgrowth cannot be attributed to a change in myelination in this model. Overall, in the slice co-cultures the degree of myelination was low, as found in the electron microscopic images at DIV 10. An earlier or later onset of myelination after treatment with GPR17 ligands might have been missed since oligodendroglial differentiation and GPR17 expression are coordinated in a temporally complex manner [43]. A longer period of cultivation and shorter intervals of measurement might show differences of myelination caused by treatment with MTK also contributing to increased neurite outgrowth.

In conclusion, the anti-asthmatic drug MTK promotes neurite outgrowth in a dopaminergic slice co-culture system. This effect can be antagonized by treatment with a new potent synthetic agonist of GPR17, pointing to GPR17 as an interesting regulator of neuroregenerative processes. Treatment with MTK results in elevated mRNA-expression of genes relevant for neuronal growth and elevated expression of the dopaminergic marker enzyme tyrosine hydroxylase. GPR17 is most abundantly expressed in NG2-positive glia, suggesting that these cell types are predominantly influenced by treatment with GPR17 ligands.

The present data are in line with previously published findings showing neuroprotective effects associated with the blockade of GPR17 in other disease models characterized by abnormal and prolonged GPR17 upregulation [12,13,24,25,65]. Moreover, the present results support that the neuroregenerative effects induced by MTK after brain injury are, in part, mediated by antagonism of GPR17 but also by additional mechanisms elevating expression of neurotrophic genes.

Repurposing of ‘old’ drugs to treat both common and rare diseases is increasingly becoming an attractive proposition, because it involves the use of de-risked compounds, with potentially lower overall development costs and shorter development timelines [69]. Intriguingly, MTK is a well-known drug that is currently part of the German guidelines for the therapy of asthma [70].

The shown putative neuroregenerative potential and its easy accessibility makes MTK an interesting drug for patients with TBI and other neurological diseases.

The data and discussions show the need for further research and development of new dual- and multi-target drugs [71], new strategies [72] and new relevant models which will help improving therapeutic strategies.

## 4. Materials and Methods

### 4.1. Materials

The following substances and factors were used: artificial cerebrospinal fluid (ACSF, composed of (mM) 126 NaCl; 2.5 KCl; 1.2 NaH_2_PO_4_; 1.3 MgCl_2_ and 2.4 CaCl_2_, pH 7.4; Hospital Pharmacy, University of Leipzig, Germany), biocytin (Sigma-Aldrich Co., St. Louis, MO, USA), dimethyl sulfoxide (DMSO, Applichem GmbH, Darmstadt, Germany), ethanol (VWR Chemicals, Darmstadt, Germany), recombinant human glial derived neurotrophic factor (GDNF; Millipore, Bedford, MA, USA), Montelukast (MTK, Biomol GmbH, Hamburg, Germany), Cangrelor (The Medicines Company, Parsippany-Troy Hills, NJ, USA).

The new potent and selective GPR17 agonists 3-(2-carboxyethyl)-4-fluoro-6-(5-methylhexyloxy)-1*H*-indole-2-carboxylic acid (PSB-16282, (PSB1), EC_50_ 12 nM) and 3-(2-carboxyethyl)-4-fluoro-6-iodo-1*H*-indole-2-carboxylic acid (PSB-16484; (PSB2), EC_50_ 32.1 nM [34]; were synthesized, purified and analysed at the University of Bonn and provided by Professor Dr. C. E. Müller (Pharmaceutical Institute, Pharmaceutical & Medicinal Chemistry, University of Bonn, Germany).

### 4.2. Animals

Rat breeding was performed in the animal facility of the Rudolf Boehm Institute, Universität Leipzig (see Institutional Review Board Statement). Rats were housed under standard conditions with free access to food and water and a 12 h light–dark cycle (lights on from 7:00 a.m.). Neonatal rat pups (WISTAR RjHan, own breed) of postnatal day 1–3 (P1–3) for the preparation of the organotypic slice co-cultures were used.

### 4.3. Preparation

The slice co-cultures were prepared from P1–3 neonatal rat pups and cultured following the “static” culture protocol as described previously [29,48,73]; for schematic illustration see Figure 1A,B). In brief, 300 µm coronal sections of mesencephalon and forebrain were cut simultaneously using two vibratomes (Leica VT1200 S; Leica VT1000 S; Nussloch, Germany). After the separation, slices of PFC and SN/VTA were transferred into petri dishes filled with 4 °C preparation medium (PM; Minimum Essential Medium (MEM), Thermo Fisher Scientific Inc., Waltham, MA, USA), adding glutamine (50 µg/mL; Thermo Fisher Scientific Inc.). The separated slices were then placed side by side as co-cultures (PFC with SN/VTA) on moist translucent membrane inserts (0.4 µm, Millicell-CM, Millipore) in a six-well plate. The wells were filled with incubation medium (IM; MEM (50%), Hank’s Balanced Salt Solution (25%), heat-inactivated horse serum (25%) (all from Thermo Fisher Scientific Inc.); supplemented with glutamine to a final concentration of 2 mM and 0.044% sodium bicarbonate (Sigma-Aldrich; pH adjusted to 7.2) and the antibiotic Gentamycin (50 µg/mL, AMRESCO, Solon, OH, USA). The cultures were stored at 37 °C in 5% CO_2_, and the medium was changed thrice weekly [73].

### 4.4. Slice Co-Culture Treatment Procedure

The slice co-cultures were kept for 3 and 10 days *in vitro* (DIV; see Figure 1C). For the pharmacological substance treatments, the slice co-cultures were divided into different experimental groups and treated with the respective substances at different concentrations (see below). The pharmacological treatment was conducted four times on DIV 1, 3, 6 and 8 while changing the IM.

The following compound concentrations were used (selected based on their potencies): 100 pM Cangrelor, 1 µM and 10 µM MTK (solved in 1% ethanol), 0.1 and 1 µM PSB-16282 (dissolved in 0.01% DMSO), 0.3 and 3 µM PSB-16484 (dissolved in 0.01% DMSO). The compounds were applied separately and in combination, to investigate if GPR17 agonists and (proposed) GPR17 antagonist could counteract their effects (to verify receptor inhibition, the antagonist was given first followed by the respective mixture of antagonist and agonist after 15 min).

The glial cell-line derived neurotrophic factor (GDNF; 50 ng/mL; solved in 1% ACSF) was used as a positive control. GDNF is known for its neural growth-promoting properties, especially on dopaminergic neurons [74]. All pharmacological substances were tested in comparison to vehicle-treated control co-cultures.

### 4.5. Fixation of the Slice Co-Cultures

To perform neurite fibre outgrowth quantification after treatment procedure (Figure 1B) and immunofluorescence labelling, co-cultures were fixed for 2 h in a solution containing 4% paraformaldehyde (Merck, Darmstadt, Germany), 0.1% glutaraldehyde (Serva Electrophoresis GmbH, Heidelberg, Germany), and 0.2% picric acid (Sigma-Aldrich) in 0.1 M phosphate buffer (PB; pH 7.4). Afterwards, co-cultures were rinsed intensively with PB. Finally, the sections were vibratome-cut into 50 µm horizontal sections.

### 4.6. Neurite Fibre Tracing Procedure

According to the previously described protocol [73], on DIV 8 biocytin crystals were placed on top of SN/VTA, incubating for 2 h allowing the uptake of biocytin and were washed with IM, subsequently.

Then, cultures were re-incubated with IM containing the pharmacological substances as previously described [29,75]. After 48 h (on DIV 10) the co-cultures were immersion-fixed (see above) and cut into 50 µm horizontal sections using the vibratome. The uptake of the anterograde tracer biocytin was labelled using the avidin–biotin complex (1:50, ABC-Elite Kit, Vector Laboratories, Inc., Burlingame, CA, USA) and the nickel/cobalt intensified 3,3′-diaminobenzidine hydrochloride (DAB; Sigma-Aldrich) was used as a chromogen. All dyed sections were transferred on glass slides, dehydrated in a sequence of increasing ethanol concentrations, and covered with Entellan (Merck, Darmstadt, Germany).

### 4.7. Neurite Fibre Density Quantification

It has been shown that dopaminergic neurons in the mesocortical projection system develop their typical innervation pattern in the organotypic slice co-cultures [29]. For quantifying neurite fibre outgrowth from SN/VTA to PFC previously described protocols have been applied [33,48,73]. Slices were used for analysis only if they fulfilled defined criteria, e.g., the slice cultures should have maintained their cellular organization, the tracer should be placed correctly on the SN/VTA, there had to be a dense network of labelled cell bodies in the SN/VTA, there had to be no labelled cell bodies in the PFC [75].

*Image analysis:* Raw images from the border region (where the two initially separated brain slices were attached) were taken in 40-fold magnification with an AxioCam ICc 1 camera (Carl Zeiss Jena, Germany) on a light microscope (Axioskop 50; Zeiss Oberkochen, Germany). For quantifying the fibre density, an automated image analysis according to a previously described technique was used [76].

After pre-processing and image binarization, the area occupied by neurite fibres was analysed. In detail, the number of pixels occupied by neurite fibres was divided by the number of pixels of the whole image, giving the percentage of the area occupied by neurite fibres called the neurite fibre density. In the shown data, one sample corresponds to the average value of the neurite fibre density (*n* = 4 slices) measured from one animal (4–9 samples (animals) were used per substance; for details see the legend of Figure 2).

### 4.8. Multiple Immunofluorescence Labelling

The free-floating slices (50 µm) were pre-incubated with a blocking solution (0.05 M Tris-buffered saline (TBS), pH 7.6), supplemented with foetal calf serum (FCS, 5%) and Triton X-100 (TX-100; 0.3%). After 30 min the slices were incubated in a mixture of primary antibodies diluted in the blocking solution for 48 h at 4 °C. The following primary antibodies were used: goat anti-glial fibrillary acidic protein (GFAP; 1:300; Santa Cruz Biotechnology, Inc., Heidelberg, Germany), mouse anti-GFAP (1:1000; Sigma), rabbit anti-GPR17 (1:100; Cayman Chemical, Ann Arbor, Michigan, USA), rabbit anti-GPR17 (1:1000; Sigma-Aldrich), goat anti-ionized calcium binding adaptor molecule 1 (Iba1; 1:100; abcam, Cambridge, UK), mouse anti-microtubule associated protein 2 (MAP2; 1:200; Chemicon International, CA, USA), goat anti-MAP2 (1:100; Santa Cruz), rat anti-myelin basic protein (MBP; 1:200; Millipore), rabbit anti-NG2 chondroitin sulphate proteoglycan (NG2; 1:200; Millipore), mouse anti-neurofilament (160 kD, NFL Medium, 1:400; abcam), mouse anti-neuronal nuclei (NeuN) (1:100; Chemicon International), mouse anti-tyrosine hydroxylase (TH; 1:1000; Chemicon International) and mouse anti-βIII-Tubulin (1:400; Promega, Fitchburg, WI, USA).

After washing the slices with TBS three times for 5 min, secondary antibodies were applied with blocking solution and incubated for 2 h. For the simultaneous visualization of the different primary antisera a mixture of the following secondary antibodies was used, specific for the appropriate species IgG (rabbit, mouse, goat). Carbocyanine (Cy2- (1:400), Cy3- (1:800), Cy5- (1:100)) conjugated IgGs; all Jackson ImmunoResearch, West Grove, PA, USA) diluted in the blocking solution were applied for 2 h at room temperature. Finally, Hoechst 33342 (Hoe, final concentration 40 mg/mL, Molecular Probes, Leiden, Netherlands) was added for nuclear staining for 5 min in TBS at room temperature. After intensive washing and mounting on glass slides, sections were dehydrated and covered with Entellan (Merck).

No immunofluorescence was observed when slices were incubated in TBS without the primary antibody.

*Image analysis:* Multiple immunofluorescence was investigated by using a confocal laser scanning microscope (LSM 510 Meta, Zeiss, Oberkochen, Germany) working with excitation wavelengths of 488 nm (argon, yellow-green Cy2-immunofluorescence), 543 nm (helium/neon1, red Cy3-immunofluorescence), and 633 nm (helium/neon2, blue Cy5-immunofluorescence). An ultraviolet laser (362 nm) was used to excite the blue-cyan Hoe 33342 fluorescence.

### 4.9. Analysis of mRNA-Expression

The tissue of the dopaminergic slice co-cultures was obtained after DIV 10. After putting the membrane inserts in 4 °C cold phosphate buffer solution (PBS; pH 7.3–7.4) PFC and SN/VTA were separated with a scalpel. Out of each well, four slices of PFC and four slices of SN/VTA were put into one tube and considered as one sample (the sample preparation was replicated four times, each using an individual animal). Then, TRIzol reagent (Life Technologies, Gaithersburg, MD, USA) was used to isolate RNA following the manufacturer’s protocol. After the first centrifugation, ethanol and GlycoBlue (Life Technologies) were added to the samples for optimal visibility of the pellets. RNA integrity and concentration were analysed with NanoDrop 1000 (NanoDrop Technologies, Wilmington, DE, USA).

The cDNA-synthesis was accomplished using the RevertAid^TM^ H Minus First Strand cDNA Synthesis Kit (Thermo Fisher Scientific Inc.) and a thermal cycler (MJ Research Inc., St. Bruno, QC, Canada). Subsequently, the samples were diluted 20-fold using distilled and RNAse/DNAse free water. Afterwards, 5 µL SYBR Green qPCR Master Mix (2X; Thermo Fisher Scientific Inc.) and 1 µL primer dilution (5 µM each) were added to 4 µL sample dilution. Then, qPCR was performed using a StepOnePlus™ Real-Time PCR System (Thermo Fisher Scientific Inc.). As reference housekeeping gene (HKG) mitochondrial ribosomal protein L32 (Mrpl32) was chosen.

The expression of the following target genes was analysed using primer sequences of growth associated protein 43 (GAP43), GPR17, interleukin-1β (IL-1β), MBP, neurofilament light chain (NFL), TH (Table 1). The following primers were supplied by Eurofins Genomics (Ebersberg, Germany): rat GAP43, rat GPR17, rat MBP, rat MrpL32, rat NFL and rat TH. Rat IL-1β was supplied by Sigma-Aldrich.

The Hot Start Polymerase was activated by a 15 min pre-incubation at 95 °C, followed by 55 amplification cycles at 95 °C for 10 s, 60 °C for 10 s and 72 °C for 10 s.

A melting curve analysis was performed to verify correct qPCR products. The following appropriate controls have been used: no template control (water) and “reverse-transcription-minus control”, in order to exclude the presence of genomic DNA in the samples. Quantification of gene expression was performed by the ΔCP method with MrpL32 serving as reference housekeeping gene. Expression levels of the respective receptors are expressed as ΔCP with the HKG MrpL32. Data are shown as bar charts (each sample *n* is equal to four slices of either PFC or SN/VTA of the respective animal).

### 4.10. Western Blot

At first, PFC and SN/VTA of cultured or native tissue were transferred into one tube, respectively. Then, 5 µL of Triton-X lysis buffer (NaCl (120 mM)), Tris (25 mM), EDTA (1 mM), TX-100 (1%), 1 mM phenylmethylsulfonyl fluoride (PMSF; Sigma-Aldrich) and protease inhibitor mix (0.5%; Sigma-Aldrich) were added to the samples at pH 7.4. The samples were homogenized, 15 min incubated on ice and then centrifuged at 5000× *g* at 4 °C. The supernatants were collected, and protein determination was performed using Pierce^TM^ BCA Protein Assay Kit (Thermo Fisher Scientific Inc.). The samples were diluted with sample buffer (1.4-dithiothreitol (DTT) (500 mM), Tris (312.5 mM), glycerol (25%), sodium dodecyl sulphate (SDS, 10%), bromophenol blue (0.005%), pyronin Y (0.005%)), pH 6.8.

This solution was cooked for 5 min at 95 °C. For performing SDS-PAGE Mini-PROTEAN Tetra Cells (Bio-Rad Laboratories GmbH, Munich, Germany) were used. At first, the 10% separating SDS polyacrylamide gel was placed in the mini cells. Before the 5% polyacrylamide stacking gel followed, 200 µL isopropanol were placed on top of the separating gel for 30 min for an ideal flat surface and were then sluiced down. The electrophoresis cells were filled with 1x Laemmli buffer (10x Laemmli buffer: glycin (1.92 M), Tris (250 mM), SDS (1%)). The calculated sample volume and 10 µL molecular weight marker (ColorPlus^TM^ Prestained Protein Ladder, Broad Range; New England Biolabs Inc., USA) were pipetted into the notches. The electrophoresis cells were connected to a power source and the gels were run for 2 h at 50 V and 400 mA.

After the gel electrophoresis, the separating gels were transferred into cathode buffer. Proteins were then transferred onto a polyvinylidene difluoride (PVDF) membrane (Westran; pore size 0.45 µm). The blotting was performed for 90 min at 1.75 mA/cm^2^. Subsequently, the PVDF membrane was stained with Ponceau S dye (Carl Roth GmbH + Co. KG; Karlsruhe, Germany) in order to make the protein bands visible.

The PVDF membrane was incubated with a blocking solution for 1 h (5% milk powder, Tris-buffered saline with Tween20 (TBST)). As primary antibody the rabbit anti-GPR17 antibody (Sigma-Aldrich; 1:1000) was used, which was normalized to β-Actin (Sigma-Aldrich; 1:5000). The primary antibody was applied in a blocking solution (5% milk powder, 0.1% NaN_3_) and incubated for 24 h at 4 °C. The secondary antibody was incubated in a blocking solution (5% milk powder, TBST) for 1 h at room temperature.

A chemiluminescent solution was applied (Super Signal West Femto Maximum Sensitivity Substrate; Thermo Fisher Scientific Inc.) and after 60 s images of the membrane were taken with a CCD camera (Diana II, Raytest, Isotopenmeßgeräte GmBH, Straubenhardt, Germany) and by using ImageJ (open source, Rasband: https://imagej.nih.gov/ij/; accessed on 8 June 2021).

### 4.11. Analysis of Cell Injury

As described previously [48], for all pharmacological treatments it was tested if they were toxic to the organotypic co-cultures by measuring the release of lactate dehydrogenase (LDH) in the IM. Briefly, the samples of IM were collected at DIV 1, 3, 5, 8 and 10 (before the cultures were fixed) and stored at −20 °C for short time. When after thawing the samples reached room temperature LDH activity was measured following the protocol described by [76]. The samples were applied to a 96-well plate format using a filter based micro plate reader device (POLAR^®^star Omega, BMG LABTECH GmbH, Ortenberg, Germany). Briefly, a sample volume of 40 µL was pipetted into the wells followed by 80 µL reaction reagent (40 µL phosphate buffer, 20 µL sodium pyruvate (1.9 mM; Sigma-Aldrich), 20 µL NADH (166.67 µg/mL; Sigma-Aldrich). Then, LDH activity was calculated by measuring the decrease in absorption of NADH at 340 nm (every 10 s for 3 min).

To exclude variations due to temperature changes between individual sets of measurements, LDH activity of all samples has been normalized to the control sample (untreated) at DIV 1 of the respective preparation.

### 4.12. Electron Microscopy

*Fixation:* For electron microscopy, rats (three 12-day-old rats) were transcardially perfused using 0.1 M PB containing 4% PFA and 1.5% glutaraldehyde (high purity, Serva). The tissue slice co-cultures were fixed with 0.1 M phosphate buffer (PB) containing 4% paraformaldehyde and 0.05% glutaraldehyde (high purity, Serva).

Fixed brains and co-cultures were further processed for electron microscopic analysis of presence of myelin-like elements. Using a Leica vibratome, horizontal sections (50 µm) of the mesencephalon were cut and the region of interest (PFC, SN/VTA) dissected. From the co-cultures of PFC and SN/VTA complex vibratome sections containing the connecting bridge between both tissue parts were performed. Selected sections were osmicated in 0.5% OsO_4_ in 0.1 M PB for 30 min, block-stained with 1% uranyl acetate, dehydrated and flat-embedded in resin (Durcupan, Fluka, Buchs, Switzerland) on glass slides. Ultrathin sections were cut by an Ultracut (Leica) and collected on single-slot Formvar-coated copper grids. Digital images of myelin-like structures/sheets in the regions of interest were taken by a transmission electron microscope Leo 906 E (Zeiss).

### 4.13. Statistics

Data are presented as mean ± S.E.M. in this study. All data were tested for normality and homogeneity of variance. The applied statistical tests are specified in the figure legends and the results (e.g., ANOVA on ranks). The probability level of 0.05 or less was considered to reflect a statistically significant difference. Significance is given as * *p* < 0.05, ** *p* < 0.01. All quantitative data have been analysed with Origin^®^ statistical analysis program (Origin^®^ 2018b, www.originlab.com/2018b, accessed on 23 July 2021).

## Figures and Tables

**Table 1:** Target genes and primer sequences.

Accession Number	Target	Forward	Reverse
NM_017195.3	rat GAP43	ACCACTGATAACTCGCCGTC	TGGCTTCATCTACAGCTTCTTTCT
NM_001071777.1	rat GPR17	ACTTGTCCTGTGTGCTGGTC	CCCAAAAGGCCAGTGATTGC
NM_031512.2	rat IL-1β	TAGCAGCTTTCGACAGTGAGG	TCTGGACAGCCCAAGTCAAG
NM_001025293.1	rat MBP	TGTGCCACATGTACAAGGACT	TTCATCTTGGGTCCTCTGCG
NM_001106116.1	rat MrpL32	TTCCGGACCGCTACATAGGTG	CTAGTGCTGGTGCCCACTGAG
NM_031783.2	rat NFL	GCAGCTTACAGGAAACTCTTGG	ACCTGCGAGCTCTGAGAGTA
NM_012740.4	rat TH	TTCTTGAAGGAGCGGACTGG	TGCATTGAAACACGCGGAAG

**Table 2:** Expression of GPR17 in organotypic slice co-cultures.

Marker	GPR17-Co-Expression
Iba1	+
GFAP	−
TH	−
NeuN	+
βIII-Tubulin	+
NFL	−
NG2	+++
O4	+
CNPase	+
MBP	+

## Data Availability

Data are contained within the article.

## Supplementary Materials

All data are available online at https://www.mdpi.com/article/10.3390/ijms222111683/s1.

## Abbreviations

ACSF	artificial cerebrospinal fluid
CysLT-R	cysteinyl leukotriene receptor
GAP43	growth associated protein 43
GDNF	glial cell line-derived neurotrophic factor
GPR17	G-protein coupled receptor 17
IL-1β	interleukin-1β
LDH	lactate dehydrogenase
LTC/D4	Leukotriene C/D4
MBP	myelin basic protein
MTK	Montelukast
NFL	neurofilament light chain
NG2	neural/glial antigen 2
NeuN	neuronal nuclei antigen
OPC	oligodendrocyte precursor cell
PFC	prefrontal cortex
PSB-16282	(PSB1) 3-(2-Carboxyethyl)-4-fluoro-6-(5-methylhexyloxy)-1*H*-indole-2-carboxylic acid
PSB-16484	(PSB2) 3-(2-Carboxyethyl)-4-fluoro-6-iodo-1*H*-indole-2-carboxylic acid
RT-qPCR	reverse transcription-quantitative real-time PCR
SDS	sodium dodecyl sulfate
SN/VTA	substantia nigra/ventral tegmental area
TH	tyrosine hydroxylase
