# Supplementary material for: Involvement of GPR17 in Neuronal Fibre Outgrowth
Source: Int J Mol Sci. 2021 Oct 28;22(21):11683. doi: 10.3390/ijms222111683 (PMC8584086; doi:10.3390/ijms222111683)
Supplement: Supplementary file 1 [file ijms-22-11683-s001.zip › ijms-1428775-supplementary.pdf]

International Journal of
Molecular Sciences

MDPI

Supplementary Materials

Involvement of GPR17 in neuronal fibre outgrowth

Max Braune[1], Nico Scherf[2], Claudia Heine[1], Katja Sygnecka[1], Thanigaimalai Pillaiyar[3], Chiara Parravicini[4], Bernd Heimrich[5], Maria P. Abbracchio[4], Christa E. Müller[3] and Heike Franke[1*]

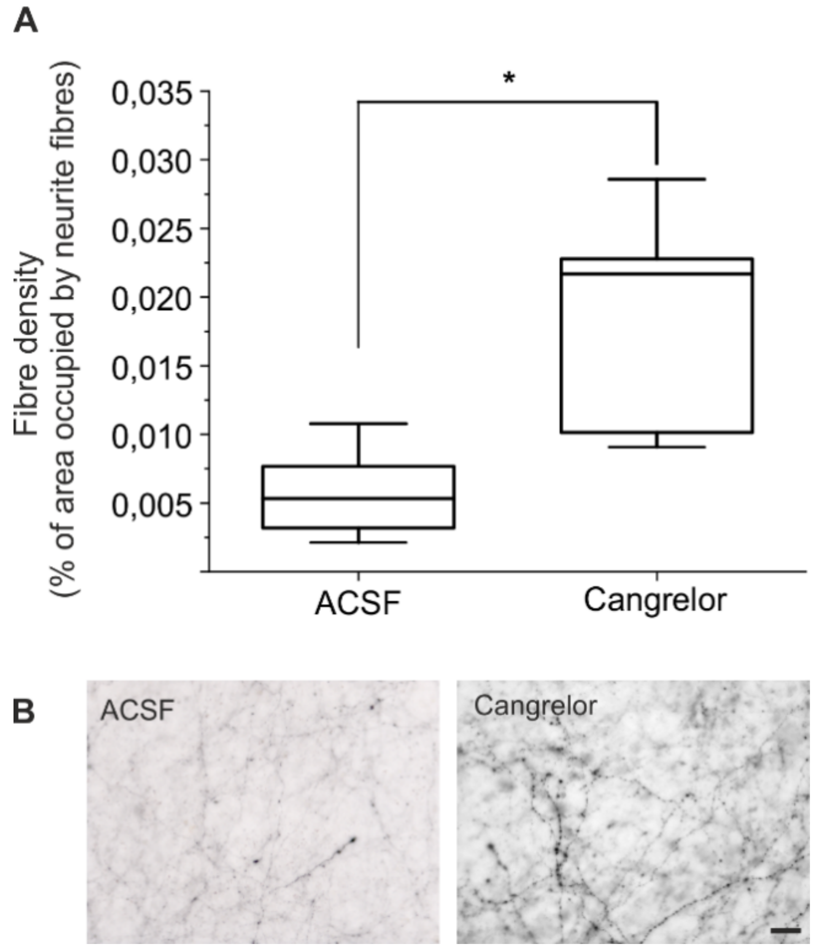

Figure S1. Neurite outgrowth quantification. (**A**) Organotypic dopaminergic slice co-cultures were treated with 0.1 nM Cangrelor and ACSF (vehicle treated control). Data are shown as box plots. The number of animals being used was n > 4. Statistical analysis was done using t-test with Welch correction t (4.34607) = -3.28534; p = 0.02685. (**B**) Pictures of biocytin-labelled fibres in the border region after application of ACSF and Cangrelor. Scale bar: 20 μm (for all).

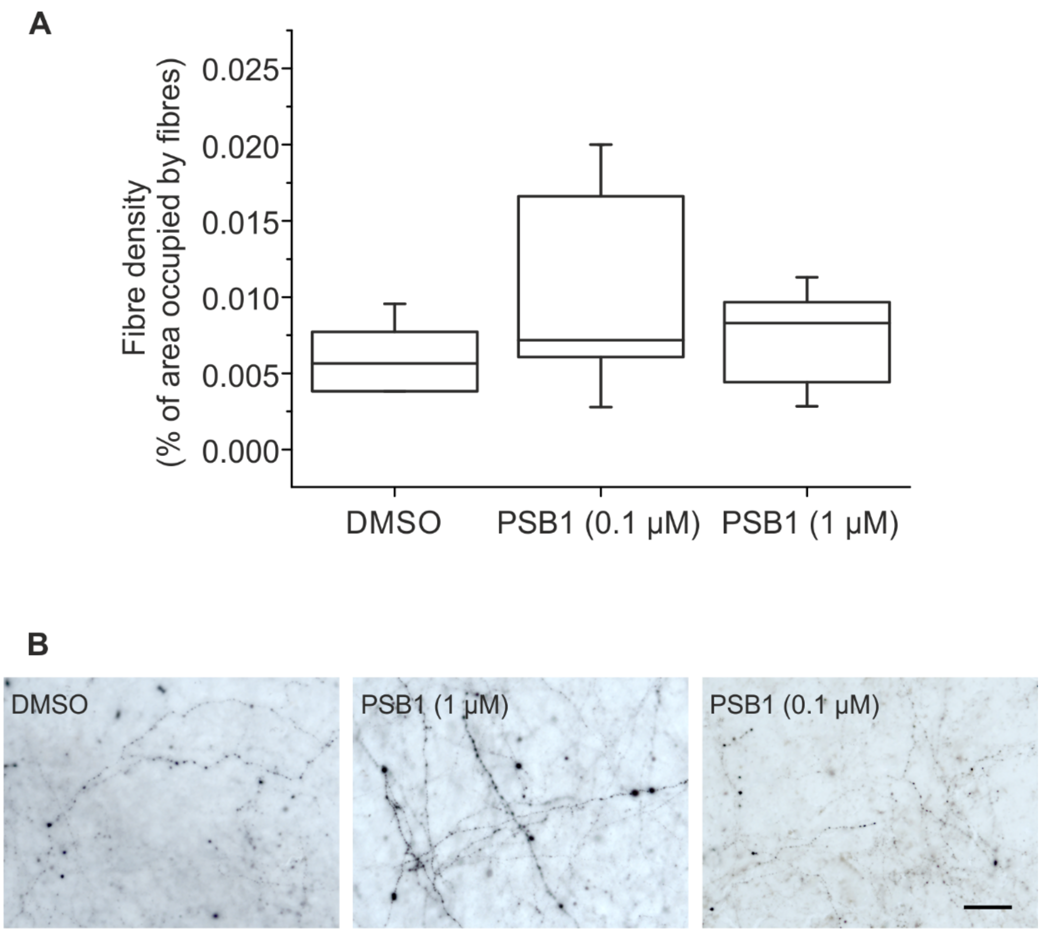

Figure S2. Neurite outgrowth quantification. (**A**) Organotypic dopaminergic slice co-cultures were treated with 0.1 µM and 1 µM PSB, respectively. All treatments were compared to vehicle treated control (0.01% DMSO). Data are shown as box plots. The number of animals being used was n⩾4. Statistical analysis was done using one-way ANOVA on Ranks. (**B**) Pictures of biocytin-labelled fibres in the border region after application of DMSO, PSB-16282 (PSB1; 1 µM) and PSB-16282 (PSB1; 0.1 µM). Scale bar: 20 µm (for all).

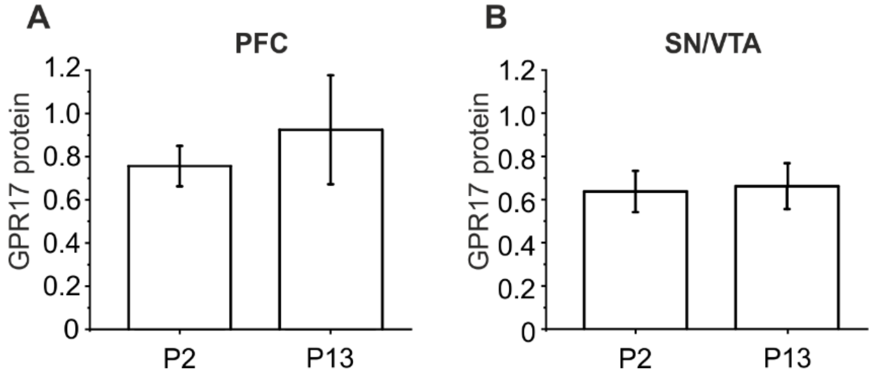

Figure S3. Results of Western blot of GPR17 *in vivo.* Protein expression of GPR17 is shown on P2 and P13 old rats in PFC (**A**) and SN/VTA (**B**), data are shown as bar charts. The number of animals being used was n ⩾ 3. In (A) for statistical analysis Mann-Whitney-Test was used, because distributions differed between both groups, Shapiro-Wilk $p < 0.05$. There was no statistically significant difference (U = 7, Z = -0,14434, p = 0,88523). In (B) for statistical analysis t-test was applied showing no statistically significant difference (t (4) = -0.15674; p = 0.88304).

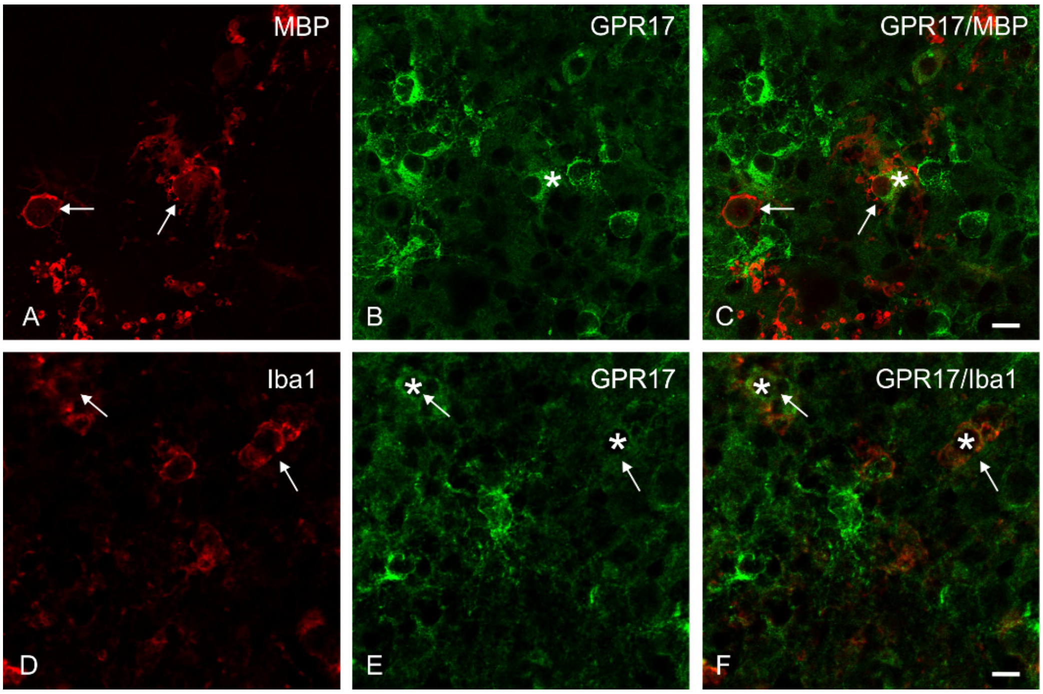

Figure S4. Multiple immunofluorescence study. Representative confocal images of GPR17 fluorescence signals in organotypic slice co-cultures. (A-C) Examples of a moderate expression of GPR17 on MBP-positive cells. (D-F) Examples of the expression of GPR17 on activated Iba1-positive microglial cells. (The stars indicate GPR17-positive cells. The thin arrows indicate the co-expression). Scale bars: A-D = 10 µm.

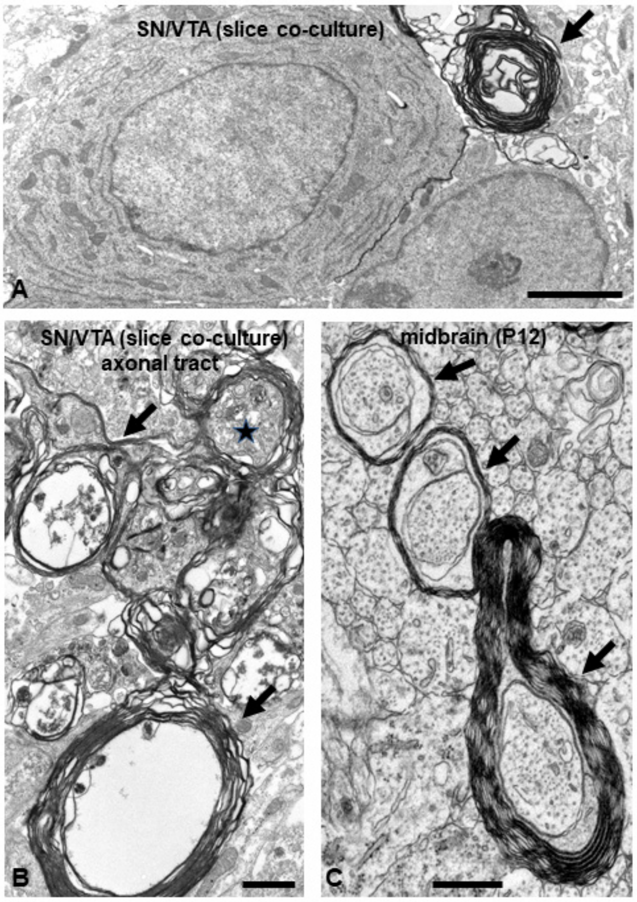

Figure S5: Electron microscopy of periaxonal myelin-like lamellae. Electron microscopy in slice co-cultures of SN/VTA and the PFC and in postnatal ventral midbrain. (A) Ultrastructural image of a myelin-like lamellar structural element (arrow) in the VTA/SN of the slice co-cultures at DIV 10. Neighboring obviously viable cell somata myelin-like lamellae of irregular appearance were observed. (B) Electron micrograph of a cross section through a fibre bridge developed between SN/VTA and PFC in the co-culture at DIV 10. Membranous-like elements varying in numbers of lamellae suggest wrapping neuronal profiles (arrows). Asterisk points to a circular lamellar rim filled with electron-dense material suggesting a neuronal profile which is undergoing degeneration. (C) Cross-sectioned axonal profiles in the ventral midbrain at P12. Axons vary in diameter and contain numerous microtubules. Most of the axons lack detectable myelin-like sheaths. Very few axons display myelin-like lamellae (arrows). Scale bar: A = 2,5 μm; B = 1 μm; C = 500 nm.
